# Influence of the Flexible Liposomes on the Skin Deposition of a Hydrophilic Model Drug, Carboxyfluorescein: Dependency on Their Composition

**DOI:** 10.1100/2012/134876

**Published:** 2012-03-12

**Authors:** Mohamed Badran, Khaled Shalaby, Abdullah Al-Omrani

**Affiliations:** ^1^Department of Pharmaceutics, College of Pharmacy, King Saud University, P.O. Box 2457, Riyadh 11451, Saudi Arabia; ^2^Department of Pharmaceutics, Faculty of Pharmacy, Al-Azhar University, Cairo 11371, Egypt

## Abstract

This study focuses on the effect of different flexible liposomes containing sodium cholate, Tween 80, or cineol on skin deposition of carboxyfluorescein (CF). Size distribution, morphology, zeta potential, and stability of the prepared vesicles were evaluated. The influence of these systems on the skin deposition of CF utilizing rat skin as membrane model was investigated. Results showed that all of the investigated liposomes had almost spherical shapes with low polydispersity (PDI < 0.3) and particles size range from 83 to 175 nm. All liposomal formulations exhibited negative zeta potential, good drug entrapment efficiency, and stability. *In vitro* skin deposition data showed that flexible liposomes gave significant deposition of CF on the skin compared to conventional liposomes and drug solutions. This study revealed that flexible liposomes, containing cineole, were able to deliver higher amount of CF suggesting that the hydrophilic drugs delivery to the skin was strictly correlated to the vesicle composition.

## 1. Introduction

The liposomes have been investigated as a systemic carriers for dermal and transdermal application of drugs [1–5]. Topical delivery of liposomal formulations may have several advantages over conventional formulations: (a) decreasing serious side-effects and incompatibilities that may happen from high systemic absorption of drugs, (b) serving as penetration enhancers, and (c) serving as a solubilization matrix [6]. Liposomal carriers have successfully enhanced the topical application of a number of drugs, including tretinoin for the treatment of acne [7], glucocorticoids for the treatment of atopic eczema [8], lignocaine and tetracaine as anesthetics [9]. There are several mechanisms that have been reported in literatures, explaining the penetration enhancement activity of liposomes. These include the vesicles that fragment at the skin surface and the vesicle components that penetrate into the intercellular lipid matrix, where they blend with the stratum corneum (SC) lipids, modifying the lipid lamellae [10, 11]. Another mechanism reported that intact vesicles could go in the SC due to the influence of transepidermal osmotic pressure [12]. Cevc et al. reported that transdermal applications of conventional liposomes have not achieved a systemic biological effect [13]. Only localized or rarely transdermal effects of liposomes have been observed.

It was reported by several researchers that the penetration enhancement of the vesicles through the skin depends on the flexibility of vesicles. Furthermore, liposomes with high flexible membranes could result in improvement of drug delivery to the skin as compared to liposomes with rigid membranes [14, 15]. Recently, sequences of new liposomes with flexible membranes were developed in order to improve the dermal or transdermal delivery of drugs, for instance, liposomes containing edge activators such as sodium cholate and tween 80 [16–18], that is, the so-called transfersomes [1, 2, 19]. Edge activators are often single-chain surfactants that destabilize lipid bilayers of the vesicles and provide flexible membrane [20–22]. It has been observed that transfersomes have penetrated the intact skin carrying therapeutic concentrations of drugs efficiently [19–22]. Moreover, the elasticity of vesicle membranes could be achieved by addition of the ethanol to the lipids. These vesicles were called ethosomes and able to penetrate the skin layers efficiently [23]. Nevertheless, due to the interdigitation result of ethanol on lipid bilayers, it was commonly supposed that the vesicles could not coexist with high concentrations of ethanol [23, 24]. Ethosomes are not plenty sufficient to convey the drugs across the SC. Therefore, a lot of approaches were developed to achieve the successful topical delivery of the drugs. Recently, novel vesicles were obtained after incorporation of ethanol and terpenes into the lipids and highly flexible vesicles were achieved. These vesicles firstly optimized by Verma et al. and used for transdermal purpose [25]. The terpenes, which are constituents of essential oils, have broadly been used as penetration enhancers. Moreover, terpenes have been proven due to their low skin irritancy at low concentrations [26]. Cineole is a terpene and extensively used as permeation enhancers with both hydrophilic and lipophilic drugs. When skin is treated with cineol, the existing network of hydrogen bonds between ceramides may get loose and break since cineol penetrates the lipid bilayer of the SC [27]. Very recently, flexible vesicles prepared using 1% cineole were described as transdermal carrier for systems [5, 28].

Therefore, the aim of this study was to prepare lipid vesicular systems with a flexible bilayer as skin drug delivery of CF as hydrophilic model drug. Usually, hydrophilic drugs are characterized by relatively poor skin penetration [29]. For this reason, three different types of flexible liposomes containing sodium cholate, Tween 80, and cineol were prepared. A further objective of this study is to obtain a deeper understanding about the mechanism of the action of the flexible vesicles containing hydrophilic drug on the skin. Liposomes were characterized in terms of particles size, zeta potential, stability, and morphology. Penetration experiments were conducted in order to investigate the penetration ability of these vesicles.

## 2. Materials and Methods

### 2.1. Materials

Soybean lecithin Lipoid S75 (Lipoid KG, Germany). 1, 8-cineole was purchased from Sigma Aldrich (Taufkirchen, Germany). Sodium cholate (Serva, Germany). Tween 80 was purchased from Sigma Aldrich (Taufkirchen, Germany). 5(6)-Carboxyfluorescein was purchased from Sigma-Aldrich, (Steinheim, Germany). Methanol (HPLC grade) was purchased from Carl Roth GmbH&Co. (Germany). Ethanol, purity ≥99.8%, was purchased from Carl Roth GmbH&Co. (Germany). Phosphate-buffered saline pH = 7.4 was composed of 136.89 mM NaCl, 2.68 mM KCl, 1.47 mM KH_2_PO_4_ and 8.20 mM Na_2_HPO_4_× 2 H_2_O (all from Merck, Darmstadt, Germany).

### 2.2. Preparation of Different Types of Liposomes

The liposomes were prepared by a conventional rotary evaporation method [30]. The lipoids S75, cholesterol, sodium cholate, or tween 80 were used (to get the optimum flexibility of the liposomes, weight ratio of surfactant and lipids should be equal to 0.28) [12, 31, 32]. Briefly, the lipids and other components (shown in Table 1) were added in a round bottom flask as solution in organic solvent, chloroform/methanol (1 : 1). The flask was connected to a rotor evaporator (Rotavapor, Büchi, Germany) and immersed in a water bath preheated at temperature equal to or more than the transition temperature of phospholipids that in the case of lipoid S75 is about 45°C. The lipid film was then flashed with nitrogen gas for removal of possible traces of organic solvents. Multilamellar vesicles (MLVs) were formed after film hydration with PBS pH 7.4 containing 20 mM CF. These MLVs were extruded through polycarbonate membranes [33] with pore size of 100 nm by means of the Avestin-Liposofast device to get liposomes of the preferred size [34].

The liposomes with the cineole were prepared by ethanol dissolution method [35]. The composition of the liposomes containing cineole is represented in Table 1. The liposomes were prepared by dissolving 1% w/v of cineol in the ethanolic solution of lipoid S75. The mixture was vortexed for 5 min and afterwards sonicated for 5 min in order to obtain a clear solution. The PBS ph 7.4 containing 20 mM CF was added to the solution by a syringe under constant vortexing. The vortexing was continued for additional 5 min. The last step was the extrusion of MLVs through polycarbonate membranes as described before.

The CF aqueous solution was prepared for comparison with vesicles system.

### 2.3. Separation of Free CF from Liposomes

The free CF was separated from entrapped by using ultracentrifugation technique (Optima Max-E, Ultra Centrifuge, Beckman Coulter, Pasadena, CA) at 50,000 rpm at 4°C, for 30 min [36]. Liposome samples were removed when the PBS was clear. Purified sediment was then diluted to the initial volume using PBS (pH = 7.4) in order to keep a final lipid concentration of 10% (w/v) and used directly for *in vitro* penetration study.

### 2.4. Physical Characterization of the Dispersions

#### 2.4.1. Particle Size and Zeta Potential Measurements

Photon correlation spectroscopy (PCS): dynamic light scattering was measured at 25°C with a Zeta plus instrument (Brookhaven Instruments, Brookhaven, USA). The samples were analyzed 24 h after preparation. The liposomes were appropriately diluted with the aqueous phase of the formulations prior to the measurements. The particle size values given are the averages of 4 measurements and are expressed as z-average. The polydispersity index (PDI) measures the size distribution of the liposomes. Zeta-potential (mV) was measured by the instrument from electrophoretic mobility of the particles [37].

#### 2.4.2. Determination of CF-Entrapment Efficiency

Free CF was separated from entrapped using ultracentrifugation method as described before. The content of CF was measured by fluorescence spectroscopy, excitation 485 nm, emission 520 nm. Entrapment efficiency of CF was calculated indirectly from the amount of free drug, according to the following equation:

(1) $$\text{Entrapment efficiency }\left(\%\right)=\left(\frac{{\text{CF}}_{t}\text{-}{\text{CF}}_{f}}{{\text{CF}}_{t}}\right)\times \text{100,}$$

where CF_*f*_ was the amount of free CF and CF_t_ was the total amount of CF.

#### 2.4.3. Storage Stability Studies

In order to determine the physical stability of vesicles, the vesicles were stored at 4°C for up to 2 months under light protection [38]. In predetermined time intervals, the particle sizes of the vesicles and PDI were measured.

#### 2.4.4. Transmission Electron Microscopy

The shape and lamellarity of the different liposomal dispersions were investigated by cryoelectron microscopy (Cryo-TEM). Five *μ*L of the dispersions were put onto a perforated coated net of copper (Quantifoil R 1.2/1.3, 400 mesh). Excess of samples were removed with a sheet of filter paper. The samples were quickly frozen with liquid ethane (−170 to −180°C) in a cryo-box (Carl Zeiss NTS GmbH). Excess of ethane was removed by blotting the samples in the cold and the samples were placed with the help of a cryo-holder (Gatan 626 Single Tilt Cryotransfer System) in a precooled transmission-cryo-electron microscope (Philips CM 120). Microscopy was performed at 120 kV and viewed under low-dose conditions.

#### 2.4.5. *In Vitro* Penetration Studies

The abdominal hair of male Wistar rat skin was removed with an electric clipper carefully. After the rats were scarified, the subcutaneous fat tissue was carefully removed from the skin using a scalpel and surgical scissors. Afterwards, the skin was wrapped into aluminum foil and stored at −20°C until use. Prior to the experiments, the skin samples were taken from the freezer and let thaw at room temperature for about 30 min. After thawing, the skin surface was carefully wiped with cotton wool balls wetted with PBS buffer. All procedures were approved by Institutional Animal Care and Use Committee and were conducted in accordance with the NIH Guidelines for the Care and Use of Laboratory Animals.

Skin samples were mounted onto Franz diffusion cells (FDCs) with a nominal area for diffusion of 1.76 cm^2^ and a receptor volume of about 12 mL. The epidermal side of the skin was exposed to ambient conditions while the dermal side was bathed with BPS buffer pH 7.4. The receptor fluid was kept at 37 ± 1°C throughout the experiments to reach the physiological skin temperature (i.e., 32 ± 1°C). The constant stirring was maintained by magnetic stirring at 500 rpm. Care was taken to remove all air bubbles between the underside of the skin (dermis) and the receptor solution throughout the experiment. After equilibration for 30 min, 10 *μ*L/cm^2^ of liposomal dispersions or PB solution containing CF were applied to the skin surface. Samples were taken from the receptor fluid (500 *μ*L) every hour and the withdrawn volume was replaced by the same volume of fresh PBS pH 7.4 to maintain a constant volume. After 6 h, the formulations were wiped off by the cotton wool pads wetted with PBS buffer 3-4 times.

For determinations of the drug deposition in the different skin layers, the skin was fixed onto cork plates and stretched using small pins. The SC was then subsequently removed by tape stripping. Transpore tape (3 M TransporeTM tape, St. Paul, MN, USA) with a surface area of approximately 4 cm^2^ was applied on the SC surface of the skin. The tape was firmly pressed on skin surface and pulled off immediately with one smooth stroke. Each skin sample was stripped with 10 pieces of adhesive tape to confirm the removal of the SC [39, 40]. The amount of CF in the stripped skin was determined by cutting into small pieces. The tapes, stripped skin were placed each in PBS pH = 7.4 : ethanol (2 : 1) overnight followed by 5 min vortexing and 5 min sonication for complete extraction of CF followed by filtration. The tapes, stripped skin, and receptor fluid were assayed for the content of CF by fluorescence spectroscopy, excitation 485 nm, emission 520 nm [5]. All experiments were done in triplicate. The extraction method was validated by spiking with a known amount of the CF. The recoveries were about 90% from tapes and stripped skin.

## 3. Results and Discussion

### 3.1. Particle Size Measurement

In the present study, three different flexible liposomes containing sodium cholate, Tween 80, and cineol were prepared to overcome the SC barrier properties. The addition of such surfactants Tween 80 and sodium cholate in vesicle membranes lead to flexibility of the vesicles membrane [40, 41]. Also, cineole is widely used to prepare flexible liposomes [5]. Recently, flexible liposomes prepared using 1% cineole as penetration enhancers with both hydrophilic and lipophilic drugs were developed [5, 42, 43]. For the efficiency of these flexible vesicles on delivering CF, a hydrophilic model drug through rat skin was investigated and compared with an aqueous solution and conventional liposomes. The compositions of these different vesicular systems, their particle size distribution, PDI, and zeta potential are presented in Table 1.

Particle size of the formulated vesicles after extruded through 100 nm polycarbonate membrane is presented in Table 1. The results showed that the average size of flexible liposomes containing Tween 80 (CF-FL2) was 133 nm with a PDI of 0.083 while, the average size of the flexible liposomes containing sodium cholate (CF-FL1) was 83 nm with a PDI of 0.051. The increasing of particle size of CF-FL2 may be due to that Tween 80 is considered as a nonionic surfactant while sodium cholate is anionic surfactants, so it had a more negative zeta potential leading to repulsion between the bilayers, so, an enlarge of the particle size of these vesicles was observed [44, 45]. In case of vesicles containing cineole and ethanol, a slight increase in the vesicle size (175 nm) was detected. The increase of the vesicle size of CF-FL3 could be attributed to presence of ethanol and cineole, which can impart negative charge and destabilize the lipid bilayer of the vesicle, which lead to an increase of the particle size. This finding is in agreement with previous study (Dragicevic-Curic et al. [42]). The particle size of conventional liposomes was 115 nm. The PDI of the investigated formulations was below 0.3, which indicates the homogeneity of the prepared CF-loaded liposomes [5, 25, 28, 42, 46].

Regarding the zeta potential measurements, all liposomal dispersions had a negative surface charge, indicating that the formulations are more stable and homogeneous distribution (Table 1). Liposomes containing Tween 80 had more negativity than sodium cholate. The reason for this result is that Tween 80 is a nonionic surfactant while sodium cholate is anionic surfactant [45]. Lee et al. revealed that Tween 80 transfersomes have a more negative zeta potential than sodium cholate. The surface charge of liposomes containing cineol recorded −65 mV. This high negative charge value of these liposomes is attributed to the presence of ethanol. Ethanol was found to increase negativity of the liposomes [24].

### 3.2. Entrapment Efficiency (EE %)

**Figure 1:**
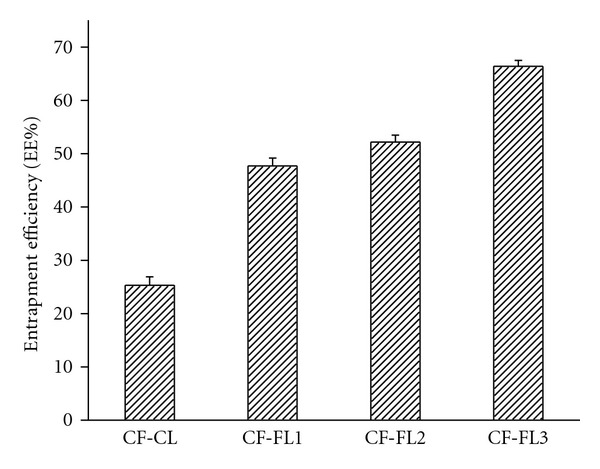
The entrapment efficiency of carboxyfluorescein- (CF-) loaded different liposomes: (CF-CL: conventional liposomes; CF-FL1: flexible liposomes containing sodium cholate; CF-FL2: flexible liposomes containing tween 80; CF-FL3: flexible liposomes containing cineole and ethanol.)

The entrapment efficiency of CF in flexible liposomes CF-FL1, CF-FL2, and CF-FL3 reported, 41.7, 52.2, and 66.7% respectively, compared with 25.3% reported for conventional liposomes CF-CL (Figure 1). It is obvious that CF-FL3 represented the largest EE% which is accompanied with increasing the particle size and zeta potential of the vesicles. A number of studies found that presence of ethanol and cineole increased the size of the vesicles and the zeta potential of liposomes [5, 28, 34, 35, 43]. The reason for that was attributed to the high value of zeta potential which frequently leads to increasing the repulsion forces of the bilayer structure of the vesicles which consequently increases the size of the inner aqueous core of the liposomes. Being CF, hydrophilic compound, increasing the size of aqueous core compartment contributes in increasing the amount of CF in the vesicles as it is observed in this study. 

Comparing the EE% of CF-FL1 and CF-FL2, the EE% of CF-FL2 was higher than that of CF-FL1 which is in agreement with other studies [43, 44, 47]. These studies suggested that differences in HLB of these surfactants may have certain impact in these findings in which the affinity of surfactants towards lipids would be inversely proportional to HLB value.

Although lower EE% is to be predictable for hydrophilic drugs, the high EE% values of such hydrophilic model in the flexible liposomes as presented in Figure 1 prove the success of the preparation method as such type of drug is expected to be restricted in the aqueous compartment of lipid vesicles [48]. The thin film-technique is supposed to get better of the entrapment of hydrophilic drugs, due to formation of a thin-film with large surface area which enables the complete hydration of the vesicles [49], which is obvious in the present study.

### 3.3. Stability Studies

**Figure 2:**
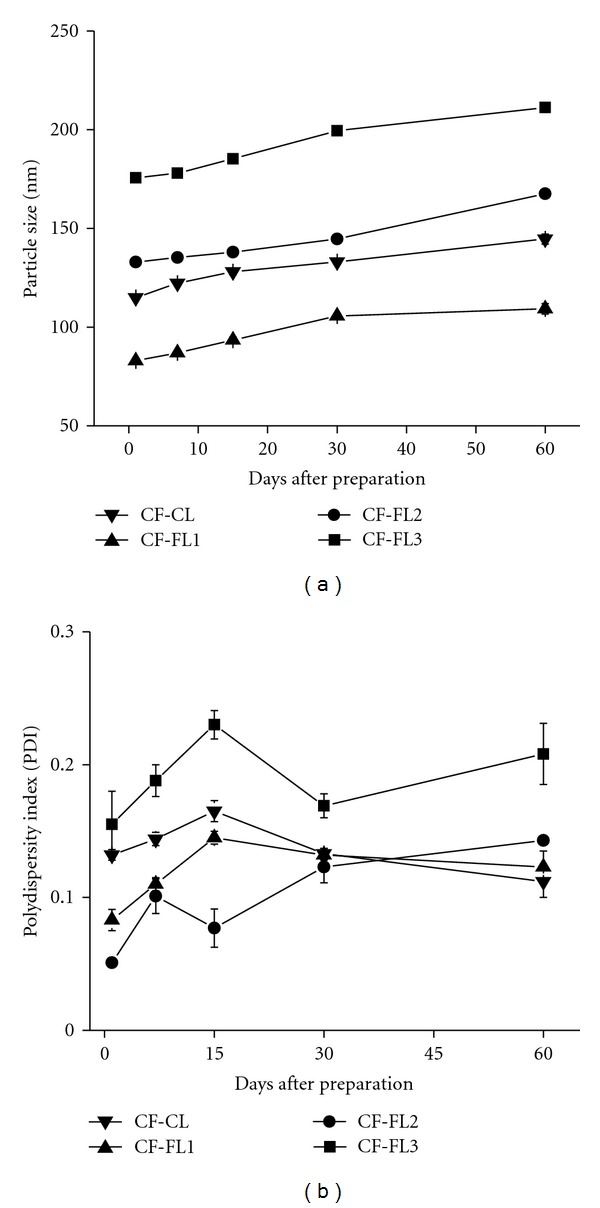
Stability study of CF-loaded liposomal dispersions stored at 4°C over 2 months duration. (a) Change of particle size (z-average), (b) change of the polydispersity index (PDI).

The physical stability of the conventional and flexible liposomes stored at 4°C for 60 days are presented in Figure 2. The stability results showed that a minimal effect on particle size and PDI of the investigated liposomes was noticed. The particle size and the PDI of different liposomal dispersion have slightly increased after 60 days storage, Figures 2(a) and 2(b). 

### 3.4. Transmission Electron Microscopy

**Figure 3:**
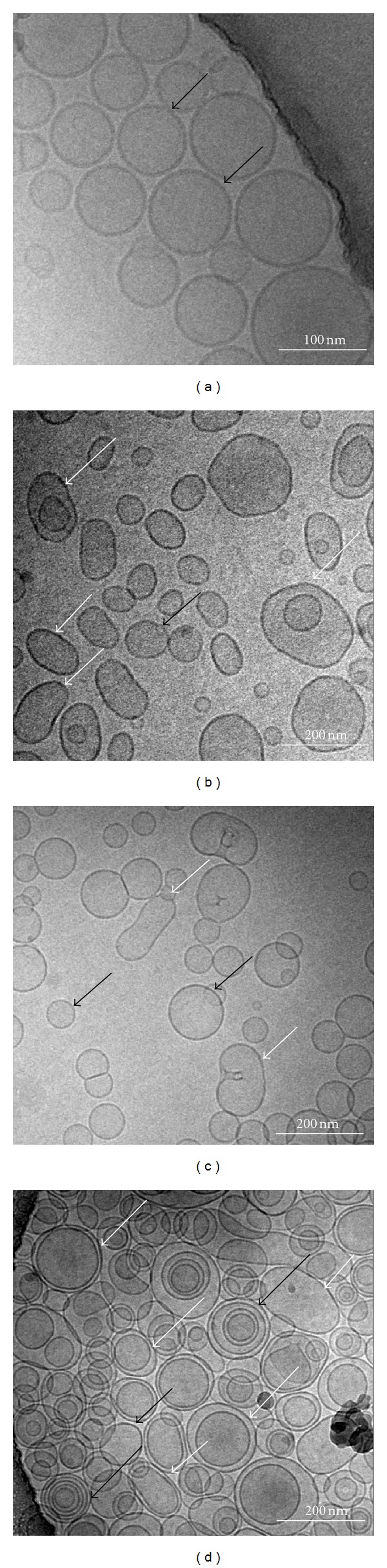
Visualization of CF-loaded liposomes by cryo-electron microscopy. (a) CF-CL, (b) CF-FL1, (c) CF-FL2, and (d) CF-FL3. Black short arrows represent unilamellar vesicles; black arrows of length represent multilamellar vesicles, white short arrows represent deformed vesicles while white long arrows represent bilamellar vesicles.

Cryo-TEM is a useful tool to identify the shapes of the vesicles. In this study, CF-loaded liposomes were mostly unilamellar (Figures 3(a), 3(b), 3(c) and 3(d) black short arrows) and bilamellar (Figures 3(b) and 3(d), white length arrows); spherical in shape; however, vesicles of irregular shapes were also detected (Figures 3(b), 3(c) and 3(d), white short arrow).

In more details, conventional liposomes showed homogeneity in size with unilamellar liposomes, also the vesicles seem to be inflexible (Figure 3(a)). In case of CF-FL1, CF-FL2, and CF-FL3 (Figure 3(b), 3(c) and 3(d)) vesicle shapes showed homogeneity in size. These vesicles seem to be flexible in comparison with conventional liposomes. These vesicles were mostly unilamellar and bilamellar, however, vesicles of irregular shape were also detected which depart from the sphere-shaped vesicles. Flexibility of these vesicles could be as a reason of the presence of cineol or edge activators (Tween 80 and Sod. Cholate) within the vesicles membrane. Presence of these compounds leads to increase deformability of the membrane (i.e., flexibility). Regarding liposomes containing cineole and ethanol, the vesicles appeared to be oval in shape and vesicles with irregular shapes were also seen. It is reported that the incorporation of cineole into the vesicles resulted in high membrane flexibility of the vesicles [28]. In addition, the liposomes with sodium cholate had lower flexibility than tween 80, due to the steroid-like structures which are bulkier than the hydrocarbon chains of Tween 80. This could be a result of their high hydrophobicity which leads to diminish the formation of transient hydrophilic holes, hence, minimizing the amphiphilic character of the bilayers responsible for membrane fluidity (flexibility) [44].

Different studies showed that the flexibility of liposomes is an important factor in the skin penetration in which more flexible liposomes enhanced drug delivery through skin in more magnitude compared with conventional liposomes [20, 21, 50].

### 3.5. *In Vitro* Penetration Studies

**Figure 4:**
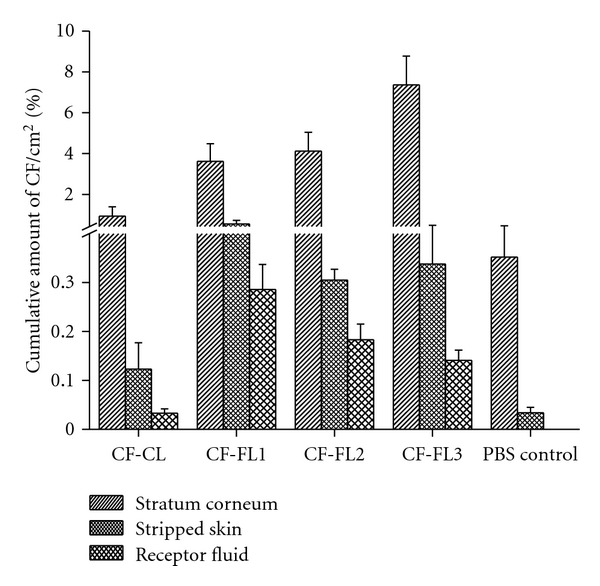
Amount of CF delivered into the SC, Stripped skin and the receptor fluid after 6 h of incubation with rat skin (CF-CL: conventional liposomes; CF-FL1: flexible liposomes containing sodium cholate; CF-FL2: flexible liposomes containing tween 80; CF-FL3: flexible liposomes containing cineole and ethanol. The controls present the PBS solution).

In order to investigate the ability of flexible vesicular systems to enhance drug deposition into the skin layers, the penetration of CF-loaded flexible liposomes and the respective PBS solution was studied and compared with conventional liposomes (Table 2, Figure 4). CF was used as hydrophilic label because a poor penetration through the skin by passive diffusion of such hydrophilic compound can be expected [51]. 

After incubation of the skin with the CF solution for 6 hours, only small amounts of CF were found in the SC, the stripped skin and the CF were not detected in the receptor fluid. When the skin was treated with the vesicular systems, particular amount of CF in the receptor fluid was detected (Figure 4). This indicates that the vesicles play an important role in the delivery of the drug into the skin layer [46]. 

Otherwise, the application of CF-CL revealed a definitely improved delivery of CF into and through the skin compared to the CF solution. The amount of CF in the SC and in the stripped skin was 2.7 and 4-fold higher for the CF-CL compared to the CF solution. Furthermore, a small amount of CF was found in the receptor fluid (Table 2, Figure 4).

Moreover, the application of flexible liposomes resulted in a further increase in the amount of CF within the skin layers as well as in the receptor fluid compared to conventional liposomes (Table 2, Figure 4). The incubation of skin with CF-FL3 showed the highest amount of CF in the SC (8-fold) and in the stripped skin (3-fold) as well as in the receptor fluid (4.6-fold) compared to conventional liposomes. CF-FL1 and CF-FL2, CF deposition into deeper skin layers (stripped skin) and the receptor fluid were pronounced but CF amounts in the SC were reduced (Table 2, Figure 4). Compared to the conventional liposomes, CF-FL1 and CF-FL2 showed an increase in the amounts of CF by 3.8- and 4.4-fold in the SC, 4.5 and 2.6-fold in the stripped skin, 9.6- and 6-fold in the receptor fluid, respectively. These results were in agreement with other studies using flexible vesicles containing Tween 80 or sodium cholate as a transdermal carrier systems [40, 50].

Interestingly, the amount of CF in the different skin layers after skin incubation with the aforementioned liposomal formulations (CF-FL1, CF-FL2, CF-FL3, and CF-CL) was varied indicating the significant impact of liposomal composition on the percutaneous absorption of compounds. These data revealed that flexible vesicles were able to deliver the encapsulated hydrophilic model drug into the receptor fluid in an amount more than that of solution [50].

Concerning the enhancement deposition of CF as a hydrophilic model drug, CF-FL3 showed higher deposition of CF in the SC than conventional liposomes and other flexible liposomes investigated in this study, indicating a potential effect of cineole and ethanol in the drug deposition. These results are in agreement with results reported by other authors who proposed that ethanol and phospholipids as well as terpenes (e.g., cineole) have a synergistic effect on fluidizing the intercellular SC lipids, which lead to enhanced drug deposition [3, 5, 25, 28, 35, 42]. Moreover, ethanol is attributed to fluidize the intercellular lipids of SC [52]. Ethanol and cineole have ability to produce flexible vesicles, which was shown by the presence of irregular shape of vesicles using cryoelectron microscopy (Figure 3(d)). Due to the flexibility of the vesicle bilayer, penetration into the SC may be facilitated particularly under nonocclusive conditions and driven by the existence of the transepidermal osmotic gradient allowing vesicles to squeeze and migrate as intact into the SC [25, 35]. Furthermore, ethanol and cineol may also act as penetration enhancer by disturbing the SC lipid structure [53, 54].

By comparing the deposition data of flexible liposomes containing Tween 80 or cholate to conventional liposomes, it is clear that the deposition of CF is high in the SC and deeper layers after application of flexible liposomes. Alternative studies proposed mechanisms that may explain the enhancement power of flexible liposomes. These proposed mechanisms include the ability of liposome to work as drug carrier systems, in which intact vesicles transported the stratum corneum carrying drug into the skin. Another mechanism stated that vesicles can act as penetration promoters, in which vesicle bilayers interact with the stratum corneum causing modification in the intercellular lipid lamellae. This effect will facilitate the partitioning of the drug molecule into and penetration through the stratum corneum [20–22]. Regarding the lower deposition of CF in the SC in presence of sodium cholate, this may be due to that sodium cholate has higher HLB (16.7) than Tween 80 (15); as a result, the affinity of Tween 80 towards lipids is expected to be high [15]. Therefore, after transdermal application of these vesicles, they follow local transdermal hydration gradient, hence, the highest CF deposition in the SC could be observed with Tween 80. A number of studies found that flexible liposomes in presence of Tween 80 increased the drug flux as compared to conventional liposomes [14, 15, 17].

Drug deposition in the different skin layers was dependent on the compositions of flexible liposomes. Skin incubation with liposomes containing cineole and ethanol led to the highest drug deposition in the SC compared to liposomes containing Tween 80 or cholate.

#### 3.5.1. Conclusion

In the present study, the flexible vesicular systems containing a hydrophilic model drug, CF along with conventional liposomes, were developed and characterized. Flexible liposomes were able to enhance the delivery of CF through skin. The highest amount of CF was deposited into the SC by flexible liposomes containing cineole and ethanol. However, the highest amount of CF was delivered to the deeper layers of the skin and receptor fluid was detected by flexible liposomes containing sodium cholate. The results of the deposition study revealed that flexible liposomes were the most effective in delivering the hydrophilic model drugs into the SC and skin layers. The flexible vesicles incorporating cineole and ethanol when applied nonocclusively onto rat skin significantly increased distribution of CF in the SC compared with conventional liposomes.

## Figures and Tables

**Table 1:** Composition of the different types of liposomes and PBS control.

Code	Lipids/components	Particle size (nm)	PDI	zeta potential
CF-CL	Lipoid-S75 10%	115 ± 1.1	0.132 ± 0.004	−12.6 ± 0.6
	Cholesterol 5%			
CF-FL1	Lipoid-S75	83 ± 3.2	0.051 ± 0.003	−26.4 ± 1.1
	Sodium cholate 2.8%			
CF-FL2	Lipoid-S75 10%	133 ± 1.4	0.083 ± 0.008	−37.4 ± 1.6
	Tween 80 2.8%			
CF-FL3	Lipoid-S75 10%	175 ± 1.2	0.188 ± 0.025	−65.3 ± 1.5
	Cineole 1%			
	Ethanol 3.3%			
PBS control	PBS (pH 7.4)			

CF-CL: conventional liposomes; CF-FL1: flexible liposomes containing sodium cholate; CF-FL2: flexible liposomes containing tween 80; CF-FL3: flexible liposomes containing cineole and ethanol; the controls present the PBS solution.

**Table 2:** Amounts of CF (expressed as cumulative % of dose applied, *n* = 3) in the different layers of rat skin after 6 hours of nonocclusive incubation.

Code	SC	Stripped Skin	Receptor	Total
CF-CL	0.93 ± 0.45	0.12 ± 0.05	0.03 ± 0.01	1.08 ± 0.17
CF-FL1	3.61 ± 0.23	0.55 ± 0.17	0.29 ± 0.05	4.45 ± 0.23
CF-FL2	4.12 ± 0.19	0.31 ± 0.05	0.18 ± 0.03	4.61 ± 0.15
CF-FL3	7.36 ± 0.13	0.34 ± 0.13	0.14 ± 0.02	7.85 ± 0.09
PBS control	0.35 ± 0.11	0.03 ± 0.01	not detectable	0.38 ± 0.04
